# What is the current state of the research literature examining the impact of the motor neurone disease journey on the couple’s relationship? A scoping review

**DOI:** 10.1017/S1478951524002141

**Published:** 2025-03-31

**Authors:** Ella Malloy, Sarah Corrie, Noreen Cushen-Brewster

**Affiliations:** 1 Institute of Health and wellbeing, University of Suffolk, Health and Wellbeing Building, Ipswich, UK; 2 School of Social Sciences and Humanities, University of Suffolk, Neptune Quay, Ipswich, UK; 3 School of Nursing, Midwifery and Public Health, University of Suffolk, Health and Wellbeing Building, Ipswich, UK

**Keywords:** MND, couple relationships, communication changes, spousal wellbeing, caregivers

## Abstract

**Background:**

Motor neurone disease (MND) results in complex and disabling symptoms that give rise to significant and challenging care needs. While much of the care required is typically provided by the partner of the individual who has been diagnosed with MND, there are few studies that have investigated the impact of MND on the couple’s relationship.

**Objectives:**

To establish the current state of the research literature examining the impact of MND on the couple’s relationship.

**Methods:**

A scoping review was undertaken with thematic analysis used to synthesize the data.

**Results:**

The scoping review identified 15 studies that were thematically analyzed to identify prominent themes. The following 5 themes were identified: adjusting to new roles; changes in communication and values; spouse well-being and health; and changes to social relationships and intimacy changes.

**Significance of results:**

This scoping review highlighted the impact of the MND trajectory on the couple’s relationship overall and on key areas of couple communication and functioning. These areas can be used to guide the development of interventions and services that are tailored to the needs of couple relationships. Further understanding of the factors impacting the couple’s relationship on the MND journey and how to navigate these factors is critically warranted.

## Introduction

Motor neurone disease (MND), also known as amyotrophic lateral sclerosis (ALS), is a neurodegenerative disease, which is ultimately fatal (Atkins et al. 2010; Kiernan et al. 2011; Leigh et al. 2003). MND has a sudden onset, usually affecting both male and female adults between 40 and 70 years of age, and has a continual deterioration. Individuals with MND develop complex and disabling symptoms, such as progressive physical disability, and cognitive and emotional changes (Atkins et al. 2010; Flemming et al. 2020; Kiernan et al. 2011; Leigh et al. 2003).

Partners of individuals with neurological conditions, particularly MND, face unique challenges due to the complex symptoms experienced. These symptoms result in complex needs that require constant care, and extensive changes to family dynamics and couple relationships following diagnosis and throughout the disease trajectory.

It is imperative that the individual with an MND diagnosis has the support of their life partner as partners are vital in supporting individuals with long-term and palliative health conditions. In fact, support from friends or other family members has been found to be incomparable to support from a partner in terms of improving psychosocial functioning while living with a chronic condition (Li and Loke 2014; Pistrang and Barker 1995). Individuals with MND are usually cared for at home, and mostly until their death, and so spouses are central to providing care to people with MND (Bruletti et al. 2015; Warrier et al. 2020). However, caring for a partner with a palliative health condition can have many negative impacts on the couple’s relationship.

Firstly, changes in the couple’s relationship can have a devastating effect on both the physiological and psychological well-being of the couple. This is because partners likely live together, meaning that the care relationship is often time-consuming and intensive (Andréasson et al. 2023; Pinquart and Sörensen 2011). The time-consuming nature of the couple’s carer relationship likely means a reduction in socialization resulting in feelings of isolation and loneliness (Li and Loke 2014). Research studies have shown that caring for a partner with a palliative health condition has a negative influence on partners’ mental health and well-being, such as an increase in depressive episodes (Burton et al. 2003). Caregiving has also been reported to affect the physical health of partner caregivers who report fatigue, less energy, and sleep disturbances (Chen and Chen 2004; Oh and Schepp 2013). Several studies have found similar findings in partners of individuals with a progressive neurological illness, such as MND (Aoun et al. 2013; Baxter et al. 2013; Conroy et al. 2021). As the MND condition worsens, there is a loss of independence and thus patients become more dependent on their caregiver, which has also been found to increase the partner’s psychological distress and worsen their quality of life (Bassola et al. 2021; Bruletti et al. 2015; Roach et al. 2009). Partners also often neglect their own health and well-being, which can lead to further declines in the partner’s mental and physical health (Li and Loke 2014).

Secondly, a diagnosis of MND can affect the dynamics of the couple’s relationship. Partners in general often feel a moral obligation to care for their partner and consequently love and caregiving roles become intertwined. This can lead to a shift in identity and autonomy with couples becoming unable to distinguish between being part of a couple and being a carer (Andréasson et al. 2023). With this change in identity, individuals can experience a loss of sense of self, which can lead to resentment. Changes in couple relationships can also lead to alterations in family dynamics as, in addition to taking on the role of carer, the spouse becomes fully responsible for housework, childcare, and financial demands, which ultimately results in changes to the power balance in the relationship (Andréasson et al. 2023).

Finally, MND comes with an array of specific, often sudden onset, cognitive and behavioral symptoms to which caregivers need to adapt (Flemming et al. 2020). Caregivers are required to master new technical and nursing skills as the condition deteriorates (Bruletti et al. 2015). Additionally, as MND develops, patients are still able to make decisions regarding their care but changes in their communication and behavior result in their partner having to become an advocate. Behavioral changes in particular have been shown to be the strongest predictor of psychosocial distress and decreased well-being of caregivers of persons with MND (De Wit et al. 2019; Olesen et al. 2022). Furthermore, as MND progresses, there are changes in intimacy and to couples’ sexual relationships due to a loss of sexual function and impairments in verbal communication, which has been found to relate to increased strain on the relationship (Atkins et al. 2010).

Research on the impact of an MND diagnosis on the couple’s relationship and the challenges faced throughout the disease trajectory is sparse. Most research to date has focused on caregiver burden, well-being, needs, resilience, and coping strategies (Warrier et al. 2020); little research has looked specifically at how couple relationships change over the course of the whole disease trajectory and the impact these changes have on both partners. Given that partners are considered vital caregivers to individuals with long-term palliative conditions and that changes in couple relationships can mutually and significantly impact both the patient’s and the carer’s quality of life and their psychological health (Li and Loke 2014; Munan et al. 2021), it is imperative to conduct research that specifically explores the changes in the couple’s relationship in order to guide future policies to help provide targeted support for the couple during these relationship changes (Flemming et al. 2020). This can be achieved through a scoping review that allows for the collating of existing research to develop new insights and identify gaps in the research, which is vital when research concerns vulnerable groups (Flemming et al. 2020).

### Research question and aims

The research question was “What is the current state of the research literature examining the impact of the MND journey on the couple’s relationship?” The aims of our scoping review were to explore

1. The nature and scope of existing research on the impact of the MND journey on the couple’s relationship.
2. The implications of the research findings and directions for future research.

## Methods

This scoping review follows the Joanna Briggs Institute (JBI) methodological guidelines for conducting scoping reviews outlined in Peters et al. (2020; 2021). In line with these guidelines, the review is reported in accordance with the Preferred Reporting Items for Systematic Reviews and Meta-Analyses extension for Scoping Reviews (PRISMA-ScR) (Tricco et al. 2018) to ensure transparency. Additionally, we followed the data extraction guidelines outlined in Pollock et al. (2023).

### Search strategy

The search strategy (Table 1) was developed collaboratively by the research team and adapted from the search strategy used by Flemming et al. (2020). Several terms for MND, carers, and relationships were used. Searches were run in the electronic databases MEDLINE, PubMed, Psychology Database, and CINAHL. Searches were run from inception to the 29th of January 2024. Screening was undertaken by a first reviewer (E.M.) and checked by a second reviewer (S.C.). From the 2787 abstracts initially reviewed, only 8 disagreements were noted. These disagreements were resolved via consensus by a third reviewer (N.C.-B.). An updated search was undertaken in May 2024.

**Table 1.** Search strategy for MEDLINE

Via Ovid, search date 29 January 2024, records identified 204
Database: Ovid Medline(R) and Epub Ahead of Print, In-Process, In-Data-Review, and Other Non-Indexed Citations, Ovid MEDLINE(R) Daily and Ovid MEDLINE(R) (1946 to present)
exp Motor Neuron Disease/
Amyotrophic Lateral Sclerosis/
(motor adj2 neuron* adj2 disease).ti,ab.
(motorneuron* adj3 disease).ti,ab.
MND.ti,ab.
Amyotrophic lateral sclerosis.ti,ab.
ALS.ti,ab.
Gehrig Disease.ti,ab.
1 or 2 or 3 or 4 or 5 or 6 or 7 or 8
Caregivers/
(caregiv$ or care giv$).ti,ab.
carer$.ti,ab.
informal care.ti,ab.
(caretak$ or care tak$ or caretaking).ti,ab.
((partner$ or spous$ or marriage) adj2 (care or cares or caring or support or supports or supporting)).ti,ab.
((husband$ or wives or wife or spouse$ or relatives or relations or families or family or familial) adj2 (care or cares or caring or support or supports or supporting)) .ti,ab.
10 or 11 or 12 or 13 or 14 or 15 or 16
Relationship$ .ti,ab.
((caregiv$ or Relationship$ or relational) adj2 (dyad or dynamic or dynamics or science)) .ti,ab.
18 or 19
9 and 17 and 20

### Inclusion criteria

The inclusion criteria for studies were as follows:

- Explores the impact of the MND journey on the relationship between partners.
- Qualitative studies, primary research, peer-reviewed material, systematic literature reviews, full-text available.
- Participant population included individuals with MND and/or partners who have experience caring for an individual with MND.
- Published in the last 20 years.
- Available in the English language.

Theses, protocols, and only abstracts available were excluded from the scoping review. Mixed methods papers were included if the qualitative data could be easily extracted.

### Data extraction

Data extraction was carried out by a first reviewer (E.M.) and checked by a second reviewer (S.C.) using guidelines outlined in Pollock et al. (2023). Relevant data included the aim, type and number of participants, methodology, results, and conclusions. The results of the data extraction can be seen in Table 2.

**Table 2.** Data extraction

	Title	Authors	Year	Country	Aim	Data type	Methods	Population	Results	Conclusion	Strengths	Limitations
1	Factors associated with grief in informal carers of people living with Motor Neuron Disease: A mixed methods systematic review	Trucco et al.	2024	US	The purpose of this mixed methods systematic review was to identify factors associated with anticipatory grief, post-death grief, and prolonged grief in informal carers of people living with motor neurone disease to inform future research and practice	Qualitative data	Mixed methods systematic review	Studies reported on carers of people living with MND. Informal carers included both current carers (those currently providing care) and former (bereaved) carers	Five overarching themes were generated through thematic synthesis: the knowledge about the progression of the disease, changes in relationships, anxiety and depressive symptoms of carers, and planning for death of the care recipient Relevant theme was relationships and changes in roles The grieving process was influenced by carers’ perceptions of the relationship between them and the person living with MND and the changes in roles in their relationship; being able to focus on the relationship with the person living with MND in a safe and relaxed context (i.e., home); and an open communication style used among family members also created a rich environment toward the end of life and emphasis on family relationships	Changes in relationships have an impact on the grieving process. In particular, changes in roles negatively affected anticipatory grief. Open communication related to a positive post-death grief process	Rigorous quality assessment, blind reviewers	A small sample of 10 studies mostly from Australia. Studies included had small datasets
2	Reflections of family caregivers and health professionals on the everyday challenges of caring for persons with amyotrophic lateral sclerosis and cognitive impairments: A qualitative study	Olesen et al.	2022	Denmark	The objective was to explore reflections of family caregivers and health professionals regarding the challenges involved in caring for persons with amyotrophic lateral sclerosis (ALS) and cognitive and/or behavioral impairments (PALS/CIs)	Qualitative data	One focus group and 10 individual semi-structured interviews were conducted with 7 family caregivers and 9 professionals after the death of a PALS/CIs	A total of 16 persons participated: caregivers (*n* = 7) and health professionals (*n* = 9), representing approximately 35 PALS/CIs. The caregivers were 2 adult children and 5 partners or spouses	Relevant themes: “Accepting that Nothing Else Matters,” “Adjusting to New Roles while Balancing,” and “Realizing Different Values in Relationships” Study reports that ALS result in changed roles in the family which required constant adjustment As the PALS/CIs became increasingly impaired, the caregivers had to take greater charge of chores, take on new responsibilities, and learn new skills Carers had to guide the PALS/CIs during public and social arrangements, where they had to deal with provocative outbursts and inappropriate behavior by their partner with ALS, which was caused by the cognitive impairments	Family caregivers found coping with the complexity of the diseases a challenge, and their everyday life needed constant adjustment to new roles, coping with inappropriate behavior, and navigating through the progression of the diseases of their sick relatives while collaborating with numerous professionals	Focus groups and interviews produced in-depth data	Small data set. Only Danish participants. Convenience sampling
3	Informal caregivers in amyotrophic lateral sclerosis: A multi-centre, exploratory study of burden and difficulties	Conroy et al.	2021	The Netherlands, England, and Ireland	The aim of this paper was to characterize and describe informal ALS caregiver cohorts attending 3 ALS multidisciplinary clinical centers (Dublin, Ireland; Utrecht, the Netherlands; and Sheffield, England) and describe the self-reported burden and difficulties associated with caregiving	Qualitative data – quantitative data not relevant to our research question	Utilized a multi-center, mixed methods descriptive exploratory study. Quantitative and qualitative data were collected during face-to-face interviews with informal caregivers from centers in the Netherlands, England, and Ireland	Seventy-six caregivers were recruited in the ALS clinic in Dublin, 58 caregivers recruited from University Medical Centre Utrecht, 38 caregivers recruited from Sheffield Clinic. Of these, 60, 49, and 32 (from Dublin, the Netherlands, and Sheffield) were spouses	Relevant themes: “Caregiver Psychological/Emotional Distress,” “Practicalities of Caregiving,” and “Caregiver Health” This study reported that caregivers experience time restrictions, are confined to the home, engage in less social activities, have more responsibility, less privacy, there is a dependency of patient on caregiver, relationship changes are prevalent	Significant differences between national cohorts were identified for burden, quality of life, and anxiety. Among the difficulties described were the practical issues associated with the caregiver role and emotional factors such as witnessing a patient’s health decline, relationship change, and their own distress	Mixed methods, large-sample size, samples are from 3 countries improving generalizability	Convenience sampling
4	Lived experience of spouses of persons with motor neuron disease: Preliminary findings through interpretative phenomenological analysis	Warrier et al.	2020	India	The objective was to explore the lived experience of spouses of persons diagnosed with MND	Qualitative data	A qualitative exploratory study with 3-point interviews was conducted with spouse caregivers of 2 persons diagnosed with MND	Spouse caregivers of 2 persons diagnosed with MND	The major themes emerged from the analysis were meaning of MND which contained the subthemes of *delay in diagnosis and deterioration, psychological response across illness trajectory*, relationship with the subthemes of *changing roles in being a carer, marital relationship, to be seen as doing;righ*t,” and *communication*; adaptation with the subthemes of *coping strategies and support system* and life without the loved one. Marital bonding between the couple, change in the relationship, and communication between the couple were described by the participants. There were 2 different opinions about the marital relationship. P1 reported that there is a change in the marital relationship; intimacy in the relationship is no longer the same. With the deterioration of symptoms, the role of the participants changed. They had to take up financial roles, and there was a change from spouse to parent. Participants narrated that they had no more time for themselves; caregiving became their primary focus and major responsibility. As a result of the multiplicity of these roles, there will be physical exhaustion, which leads to further emotional distress and frustration	There are changes in the lives of spouses and in strategies for caring the partner with deterioration of symptoms in the illness trajectory. The palliative approach in the management of MND has to take into account the experiences and needs of carers since care happens at home	Semi-structured interviews produced in depth data.	Small sample, language barriers noted
5	The impact of respite care from the perspectives and experiences of people with amyotrophic lateral sclerosis and their care partners: A qualitative study	Wu et al.	2022	Canada	The aim of this study was to explore the impact of respite care through the perspectives and lived experiences of people with ALS and their care partners	Qualitative data	Participants were assigned to either the control group or the respite care intervention. Respite care was provided in the form of home-based services. Semi-structured interviews were conducted with participants at baseline and after a 6-month period	Thirty-one dyads (62 participants) of people with ALS and their care partners	Caregiving challenges specific to the care partner and the patient-care partnership relationship were identified. Overall, people with ALS and care partners responded positively to in-home respite care and reported improved relationship quality, more time for the care partner to pursue personal commitments or take a break, and improved emotional well-being for both the person with ALS and the care partner. Barriers and concerns were raised surrounding privacy and staff consistency	This study highlights respite care as a critical tool to alleviate caregiving challenges and support the needs of people with ALS and their care partners. Engagement with the ALS community and formal evaluations of respite care services should be prioritized to minimize barriers and best meet the needs of people with ALS and their care partners	Valid scales, mixed methods, semi-structured interviews	Small sample. Lack of ethnic diversity
6	Transitions in amyotrophic lateral sclerosis: Patient and caregiver experiences	Munan et al.	2020	Canada	The objective was to understand how Person with ALS (PwALS) and caregivers experience transitions throughout their ALS journey	Qualitative data	Semi-structured interviews were audio-recorded, transcribed, and analyzed using qualitative thematic analysis	PwALS and their caregivers were recruited from a multidisciplinary ALS clinic in Edmonton, Canada. We recruited patients at the stage of ALS where home mechanical ventilation, a feeding tube, and/or assistive communication technology had been offered. Fourteen PwALS and 15 caregivers	The importance of community was identified by many PwALS and caregivers who expressed feelings of loneliness and isolation. Most caregivers were spouses and couples navigated a change in their relationship roles as 1 spouse transitioned to becoming a caregiver, while the other transitioned to dependency. The caregiver spouses reported a sense of “total responsibility” that encompassed continual vigilance for the PwALS’s well-being, managing their household and finances. PwALS and caregivers reported transitioning to reliance on life-sustaining medical devices; early adoption and information on these devices increased their quality of life. Participants also wanted more and earlier information on advanced care planning. PwALS and caregivers identified adapting to new forms of communication as a necessity	ALS presents many transitions for PwALS and caregivers. Understanding these transitions is important for ALS health-care professionals who seek to implement best-care practices	Large inclusion criteria so heterogeneous sample. Interviews conducted in participants’ home	Participants were recruited from only one ALS clinic. Cross-sectional
7	Going inside the relationship between caregiver and care-receiver with amyotrophic lateral sclerosis in Italy, a grounded theory study	Bassola et al.	2019	Italy	The objective was to investigate the relationship between ALS patients and their family caregivers and how it impacts care, and patient and caregiver outcomes	Qualitative data	Interviews	Twenty-two patients/caregivers in a neuromuscular clinical center in the South of Europe	From the interviews, 3 main categories emerged: “reciprocity,” “loving to care,” and “changing to care,” and 4 secondary categories: “having support,” “sharing suffering,” “protecting each other,” and “thinking positive”	A stable and calm relationship between patient and caregiver, characterized by reciprocity, mutual help, and affection, affected patient self-care provided at home and the caregiver's burden	Semi-structured interviews	Mono-centric study. Small numbers of participants
8	Caregiving in ALS - a mixed methods approach to the study of burden	Galvin et al.	2016	Ireland	The purpose of this analysis was to describe an informal caregiver cohort, their subjective assessment of burden, and difficulties experienced as a result of providing care to people with ALS	Qualitative data – quantitative data not relevant to our research question	Mixed methods – Semi-structured interview and open-ended questionnaire	Eighty-one participants. A majority of this caregiver cohort was female (70%) and spousal caregivers (72%)	From the qualitative data, the caregiving difficulties were thematized around managing the practicalities of the ALS condition, the emotional and psychosocial impact, limitation and restriction, and impact on relationships	This study explored the complexity of caregiver burden in ALS. Understanding the components of burden and the difficulties experienced as a result of caring for someone with ALS allows for better supporting the caregiver, and assessing the impact of burden on the care recipient	Semi-structured interviews	Sample only from one clinic
9	Factors that facilitate and hinder the manageability of living with amyotrophic lateral sclerosis in both patients and next of kin	Olsson Ozanne et al.	2011	Sweden	This study aimed to illuminate factors that facilitate and hinder the manageability of living with ALS in patients and next of kin	Qualitative data	Interviews	Fourteen patients and 13 next of kin	The results indicate constant fluctuation between opportunities and limitations in the individual ability – of patients and family members – to manage the life situation. Both patients and next of kin devised strategies to manage their situations through acceptance, living in the present, and perceiving real presence and support from family, friends, and authorities. Dysfunctional relationships with family members, friends, or authorities reduced the manageability of the situation. Furthermore, patients experienced difficulties managing their situations when forced into passivity and increased dependence. Next of kin experienced decreased ability to manage because of burden, lack of own time, and feelings of being controlled	The fluctuations in manageability and the similarities and differences between the pairs indicate the importance of support, both for the individual and the family	Semi-structured interviews	The study was limited in that it was necessary to divide the material into 3 content areas based on the SOC components of comprehensibility, manageability, and meaningfulness and then focus solely on manageability because of the extensive interview data
10	Needs of informal caregivers across the caregiving course in amyotrophic lateral sclerosis: A qualitative analysis	Galvin et al.	2018	Ireland	The objective of this analysis was to explore the needs of informal ALS caregivers across the caregiving course	Qualitative data	Semi-structured interview, which took place on 3 occasions at 4- to 6-month intervals	Home interviews at baseline (*n* = 81) and on 2 further occasions (*n* = 56, *n* =41) with informal caregivers of people with ALS attending the National ALS/MND Clinic at Beaumont Hospital, Dublin, Ireland. Fifty-eight spouses	The majority of caregivers were family members. Hours of care provided and caregiver burden increased across the interview series. Thematic analysis identified what would help them in their role, and needs related to external support and services, psychological–emotional factors, patient-related behaviors, a cure, and “nothing.” Themes were interconnected and their prevalence varied across the interview time points	This study showed the consistency and adaptation in what caregivers identified as helpful in their role. Support needs are clearly defined and change with time. Caregivers need support from family, friends, and professionals. Identifying the specific needs of informal caregivers should enable health professionals to provide tailored supportive interventions	Longitudinal study	Loss of participants over time could have caused bias
11	Experiences of burden, needs, rewards and resilience in family caregivers of people living with Motor Neurone Disease/Amyotrophic Lateral Sclerosis: A secondary thematic analysis of qualitative interviews	Weisser et al.	2015	England	The aim of this study was to explore the experiences of family caregivers of people with motor neurone disease/ ALS, specifically the relationship between positive and negative experiences of caring and to identify possible ways to better support these caregivers	Qualitative data	Secondary thematic analysis of 24 semi-structured qualitative interviews conducted longitudinally	Ten family caregivers	Themes emerged around burdens, needs, rewards, and resilience. Resilience included getting active, retaining perspective, and living for the moment. Burden was multifaceted, including social burden, responsibility, advocacy, ambivalence, guilt, and struggling with acceptance. Rewards included being helped and “ticking along.” Needs were multifaceted, including social, practical, and psychological needs. The 4 main themes were interrelated	Burden, resilience, needs, and rewards are interrelated. Caregivers’ ability to cope with caring for a person with motor neurone disease/ ALS oscillates between positive and negative aspects of caring, being at times active, at times passive	Semi-structured interviews. Mixed methods	Small-sample size. Interviewed participants over the phone
12	Psychoeducational groups for people with Amyotrophic Lateral Sclerosis and their caregiver: A qualitative study	Bilenchi et al.	2022	Italy	The current study aimed to describe the implementation of a structured psychoeducational intervention in ALS, identifying the needs of both patients and their caregivers	Qualitative data	Five patients and 13 caregivers attended 8 psychoeducational group meetings. Eight participants underwent semi-structured interviews once the group sessions ended	Five patients and 13 caregivers	Seven main themes were identified: “practical advice,” “explanation of the pathology,” “recognition of emotions,” “adaptation,” “family and relationships,” “being caregiver of oneself,” and “sharing”	This study displays the utility of psychoeducational group intervention in supporting people with ALS and their caregivers because of 2 main reasons: first for the psychoeducation provided by professionals; second for the possibility of sharing experiences and emotions with people in the same situation	In-depth data from interviews and group meetings	Small sample, no control group
13	The experiences of, and need for, palliative care for people with motor neurone disease and their informal caregivers: A qualitative systematic review	Flemming et al.	2020	England	To explore the experiences of, and need for, palliative care of people with motor neurone disease and their informal carers across the disease trajectory	Qualitative data	A systematic review of qualitative research conducted using thematic synthesis	A total of 41 papers were included, representing the experiences of 358 people with motor neurone disease and 369 caregivers	Analytical themes were developed detailing patients’ and carers’ experiences of living with motor neurone disease and of palliative care through its trajectory including response to diagnosis, maintaining control, decision-making during deterioration, engaging with professionals, planning for end-of-life care, and bereavement	The review identified considerable literature exploring the care needs of people with motor neurone disease and their carers; however, descriptions of palliative care were associated with the last days of life. Across the disease trajectory, clear points were identified where palliative care input could enhance patient and carer experience of the disease, particularly at times of significant physical change	Vigorous literature review	The number of studies included in the review was “actively managed” through purposive sampling
14	“Who will I be?”: Relational identity, living with amyotrophic lateral sclerosis, and future-oriented decisionmaking	Versalovic et al.	2020	US	NA	Qualitative data	Interviews	NA	This study highlights 2 critical aspects of the patient–caregiver relationship: (1) the extent to which each may rely on the other leaves their well-being intimately intertwined and (2) patients often require others to help with the imaginative task of considering possible futures for each therapeutic option. This study shows why family involvement in decision-making practices can be so critical, and sheds light on the ways intimate others help preserve and protect people’s identities amidst the destabilizing uncertainty illness and treatment can bring	NA	NA	NA
15	Experiences with advance care planning in amyotrophic lateral sclerosis: Qualitative longitudinal study with people with amyotrophic lateral sclerosis and their family carers	Vandenbogaerde et al.	2024	Belgium	The aim of this study was to understand the experiences with advance care planning of persons with ALS and their family carers – and if, when, how, and why these experiences change over time	Qualitative data	Interview	Nine persons with ALS and 9 family carers	All participants thought about future care, but few talked about it. Over time, advance care planning experiences were influenced by intertwined elements: (1) experienced physical decline and related future care needs; (2) how persons with amyotrophic lateral sclerosis identify themselves as patients; (3) obtaining information about diagnosis and prognosis; (4) professionals initiating conversations about medical aspects of end-of-life decisions; (5) balancing between hope to remain stable and worry about the future; and (6) protecting themselves and each other from worries about the future	This study emphasizes how factors such as coping with the disease and relational dynamics shape individuals’ thoughts about future care over time and how psychological, social, and medical factors are interwoven in advance care planning	Longitudinal study, in-depth qualitative information	Participant selection bias No follow-up interview, Small number of participants

### Synthesis

A thematic analysis approach was used to synthesize the data. We utilized the thematic analysis framework developed by the National Centre for Social Research (Ritchie and Spencer, 2002). This framework involves a systematic approach consisting of several phases. First, the researchers familiarized themselves with the data, and codes were generated based on the research question. A thematic framework was then established by revisiting the aims of the study while also identifying any emerging themes; from this process, several subthemes also emerged within the overarching themes. Each theme was then clearly defined to reflect the patterns in the data. Differences in coding were resolved by consensus among the research team, more details can be seen in the Online Appendix.

### Results of search and inclusion

**Figure 1.**
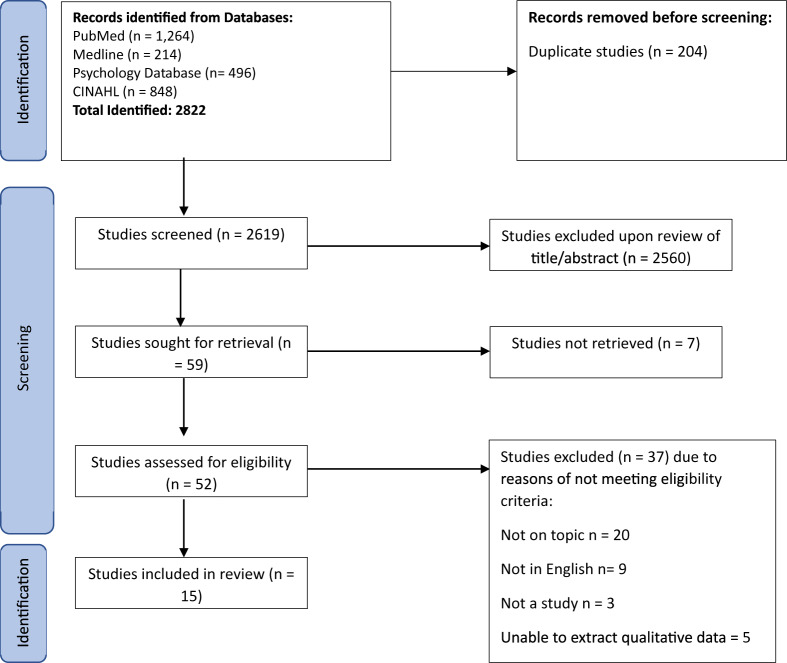
PRISMA-ScR 2020 flow diagram.

A total of 2822 results were identified from the electronic databases. Following removal of duplicate studies, 2619 studies were excluded based on title and abstract screening (see Figure 1). A total of 52 studies remained for full review, of which 15 were found to be relevant to the aims of the scoping review and fit within the inclusion criteria of the search.

### Characteristics of included studies

The majority of the studies were published in the United Kingdom (2), the United States (2), Ireland (2), Italy (2), and Canada (2). Other studies were published in Denmark (1), India (1), Sweden (1), and Belgium (1). One study included a sample from Ireland, the Netherlands, and England. All but 1 of the studies was published in a peer-reviewed journal. Most of the included studies focused on patient and carer dyads (7) or just carers (6), while 1 study focused on health-care professionals and carers. Most of the studies were qualitative (11). The remaining studies were mixed methods (4).

### Characteristics of participants

The experiences of 358 individuals with MND and 560 carers were represented. The age range of individuals with a diagnosis of MND was 25–84 years. Of the studies which reported participant characteristics, most of the individuals diagnosed with MND were male (250; 181 female). The age range for the carers in the included studies was 22–81 years. Most carers were female (241) compared to male (194). The experiences of 253 partners or spouses were represented in the studies with the remaining carers represented being made up of family members, informal carers, or having unspecified relationships to the individuals with MND. For consistency, spouses and partners will be referred to as partners throughout the paper.

### Thematic analysis

Five themes were drawn from the review of the study findings. These themes were broken down further into several subthemes:

1. Adjusting to new roles
  1. New household roles
  2. Role as carer
2. Changes in communication and values
  1. Communication issues
  2. Changes in personal values
3. Spouse well-being and health
  1. Psychological well-being
  2. Physical well-being
4. Changes to social relationships
5. Intimacy changes

The numbers in superscript relate to the corresponding number assigned to the studies in Table 2.

### Adjusting to new roles

#### New household roles

The findings of the studies suggested that as the disease progresses and the range of impairments increases, partners of individuals with MND had to take on more roles and responsibilities, such as financial responsibilities, and often experienced a role reversal.^1,2,3,4,5,6,7,8,10,12,13^ Often following a diagnosis of MND, the household income reduced from 2 incomes to 1, the partner had to reduce their working hours, or stop working altogether to meet the demands of the carer role, which resulted in less money and financial concerns.^4,5,6,10,13^ Additionally, some studies revelated that partners had to learn new skills related to the pragmatics of daily life, such as mastering household tasks, home repairs, or car maintenance, which were previously the role of the person diagnosed with MND.^3,6^ Additionally, several studies reported how following a diagnosis of MND there was often an alteration in family dynamics, with partners having to take on more parental responsibilities.^2,4,5,8,10,12^ These shifts in household and family roles often caused stress and frustration which at times resulted in conflicts within the couple’s relationship.^2,5,6^

#### Role as a carer

In addition to having to adjust to new household roles, partners also reported having to adjust to becoming a carer. Several studies reported that individuals with MND became completely dependent on their partner as their condition progressed.^3,5,6,9,15^ Partners felt completely responsible for their partner’s health and believed that they had a duty of care to their sick loved one.^6,8,11,13,15^ The studies reported that undertaking the role of a carer led to feelings of immense pressure from having to take on too much/total responsibility for their partner resulting in feeling as though their own life had become restricted or put on hold.^3,5,6,8,13,15^ As the condition worsened and dependency on medical devices increased, partners had to learn new skills to adapt to their new role as a carer such as learning how to use wheelchairs, eye gaze systems, and Percutaneous Endoscopic Gastrostomy (PEGs).^2,3,6,8,15^ They also had to provide physical support such as helping their partner to get dressed, transferring them to beds or wheelchairs, and guiding them through their daily activities, which they reported as difficult to adapt to.^2,5,8,13,3^ Additionally, as the partner took on the role of carer, they also became an advocate for their spouse and an important source of information. This resulted in them becoming responsible for making decisions and for their partner’s health-care needs and visits.^2,11,13,14^ These changes in roles made partners feel more like a parent or carer than a spouse, which led to changes in their relationship dynamics.^4,5,8,15^

### Changes in communication and values

#### Communication issues

Several studies reported changes in communication to be an issue in couple relationships as a function of MND inhibiting speech.^2,4,7,8,10^ Specifically, partners had to develop new forms of communication such as using eye gaze equipment, voice banking software, or simpler methods such as hand signals, which, while improving the patient’s quality of life, led to frustration and misunderstandings.^6,8,13^ Several studies also reported that communication issues led to partners feeling lonely as they were unable to have the same level of connection that they once had with their partner.^6,8,9^ Patients reported still wanting to be in control of making decisions; however, with a decline in communication skills this became complex and distressing and often resulted in conflicts between partners.^2,6,8,13,15^ Some studies reported that both partners often hid their feelings as they did not want to upset or burden the other person.^9,12,13,15^ However, interestingly, other studies reported that open communication is important within the couple’s relationship, with mutual understanding and having an open, reciprocal relationship prior to diagnosis helping to reduce frustration and burden during the disease trajectory.^1,7,9,10,15^

#### Changes in personal values

In addition to changes in communication, studies also reported that partners’ personal values changed.^1,2,4,7,9,11,13^ In particular, several studies reported that as time together was now limited, spouses experienced a change in their outlook and values such as prioritizing living in the moment and appreciating what little time they had left, learning to be resilient and how to have a positive outlook, as well as putting their own life on hold to engage in activities that mattered to their partner.^2,7,9,13^ However, some spouses reported that this led to changes in their identity and stated that they experienced a loss of self as they had to reinvent themselves to adapt to their new situation.^1,7,13,15^ Other studies suggested that partners experienced difficulty accepting the diagnosis at the beginning which had a negative effect on their values and outlook.^1,4,9,11,13,15^

### Spouse well-being and health

Most studies reported that caring for a partner with MND had a significant effect on the caregiver’s physical and mental health and well-being.

#### Psychological well-being

The studies reported that partners experience a range of complex and ever-changing emotions caring for their partner with MND. These included fear, anger, sadness, and frustration due to changes in their partner’s behavior and language and uncertainty about the future.^1,2,3,4,5,8,9,12,13,15^ Partners frequently experience psychological and emotional distress from watching someone they love deteriorate.^3,8^ They also reported feeling a sense of loss while their partner was still alive due to changes in their behavior and language, and no longer appearing to be who they once were.^2, 8, 13^ Partners reported experiencing guilt and conflicting emotions for a number of reasons including being in love with their partner but often hoping that the MND journey would come to an end due to the immense burden they felt, feeling fearful of the future but wanting to live in the moment, and feeling sorry for their partner but also angry because they missed their former life together.^2,4,5^ The studies reported that caring for a partner with MND did not allow time to oneself to pursue hobbies or take a break from care responsibilities which negatively impacted the carer’s well-being and led to frustration. ^3, 4, 5, 8, 9, 10, 12^ Some studies reported that respite care is important in giving partners time to themselves, which was found to improve their well-being and the quality of the couple’s relationship. However, partners were often unwilling to utilize respite care as they felt totally responsible for their partner and did not want to relinquish control over their care.^5,8,10^ In addition to feeling the burden of responsibility, studies reported that partners often felt as if they were not doing enough as they could not aid recovery, which led to feelings of helplessness.^5,6,13^ Partners were reported as often neglecting their own mental health and well-being in favor of adopting the role of carer.^2,5,10^ Several studies reported that there is currently a lack of support for partners of individuals with MND.^1,14,13^

#### Physical well-being

The studies reported that partners typically had little or even any time to themselves, which led to reports of physical exhaustion and poor sleep.^2,3,4,5,13^ Additionally, partners often reported becoming injured as a result of the physical demands of caring, such as experiencing strain and back injuries from lifting as their partners mobility reduced throughout the disease trajectory.^3,8,13^ The physical exhaustion and injuries the partners experienced were further exacerbated by neglecting their own needs and health. Partners reported not taking the time to themselves to address their own health problems or needs, which frequently led to them getting sick, an increase in comorbidities, and canceling or missing doctor appointments.^3,4,5^

### Changes to social relationships

Studies reported that partners of an individual with MND experienced changes in their social relationships. These changes occurred for several reasons. First, partners reported having a lack of time to socialize as well as being confined to the home, which limited their ability to engage in regular activities, make plans, or respond spontaneously to invitations. This reduced the number of social relationships that partners were able to maintain resulting in a loss of friendships.^5,6,8^ Second, due to cognitive impairments, individuals with MND can be prone to emotional outbursts and display inappropriate behaviors in public which partners reported led to feelings of awkwardness or even confrontations with other members of the public. These experiences left partners feeling more hesitant to leave the house.^2,13^ Third, while studies reported that both social and family support are important, partners of individuals with MND often felt like a burden to their family and felt guilty asking their family for help so took on total responsibility of care.^7,9,12^ Leading on from this, some studies reported that partners were unwilling to share the burden of care with others and did not want outside help.^3,5,10,11,13^ Partners reported becoming frustrated with the increase in people coming into home (i.e. community health-care workers).^2,5,13^ The loss of social relationships impacted how caregivers related to other people and themselves^1,2,3,4,6,8,13^ and led to feelings of loneliness and a sense of being trapped.^1,2,9,12^

### Intimacy changes

The final theme identified from the studies relates to changes in intimacy. There was a limited number of studies that reported on intimacy changes in relationships, with one study suggesting this is because the topic is rarely discussed.^13^ However, the studies that reported on intimacy changes suggested that due to physical impairments, intimacy between couples was reduced or significantly altered. Partners reported that as the condition deteriorated, they could still kiss and hug their partner, but increasingly their partner was only able to passively respond.^2,5^ Several studies reported that there was a reduction in relationship satisfaction and sex life as the disease progressed.^2,4^ Despite changes to intimacy, spouses in several studies reported that love and mutuality was important during the disease progression, with strong prediagnosis marital relationships and a shared love for one another enabling mutual comfort which reduced the burden on the carer and resulted in the relationship staying strong after the MND diagnosis.^7^

## Discussion

Our review sought to identify, examine, and synthesize the qualitative evidence on the current state of the research literature examining the impact of the MND journey on the couple’s relationship. The included studies reported on the impact of a partner diagnosed with MND on role adjustment, changes in communication and values, partner well-being, social relationships, and intimacy.

Most of the studies reported on the impact of MND on role reversal and the adjustment of the couple’s relationship to new household and caring roles. These shifts in household roles and family dynamics can lead to feelings of frustration, stress, and conflict within the couple’s relationship (Olesen et al. 2022). Couples often experienced identity issues due to this change in their relationships, with individuals with MND no longer able to take on roles they were once responsible for and their partners no longer seeing themselves as a spouse but as a carer (Pinto et al. 2021). With this change in the couple’s identity, partners can experience a loss of self, which can lead to resentment and changes in the balance in the relationship (Andréasson et al. 2023). These changes are further exacerbated by having little time to themselves and due to the timeframe of the disease trajectory (Conroy et al. 2021). This supports research which found that partners experience a sense of moral obligation (“for better or worse”) to care for their spouse. The new caregiving/care-receiving relationship that forms change the dynamics of power in the relationship as well as the partner’s personal autonomy (Andréasson et al. 2023).

Communication was a key theme that emerged from the scoping review, particularly in relation to how this impacted the couple’s relationship. MND leads to a degeneration of both upper and lower motor neurones, which can cause impairment in communication, breathing, and swallowing (Paynter et al. 2019). The studies reported that couples had to develop new forms of communication with each other. However, this often led to frustration, misunderstandings, and feelings of intellectual and emotional isolation, as communication reduced (Paynter et al. 2019). Obviously, this reduction in communication influences the couple’s relationship and their sense of connectedness. Furthermore, as communication skills deteriorate, the partner with MND becomes increasingly reliant on their partner’s support which, as previously stated, changes the couple’s relationship dynamic (Andréasson et al. 2023; Paynter et al. 2019). Joubert and Bornman (2012) suggest that maintaining communication is vital in dealing with emotions elicited by changes caused by MND. Alternative communication strategies can help to maintain intimate relationships between couples such as communication aids and sign language (Joubert and Bornman 2012).

A further theme that consistently emerged from the review was the impact that changes in the couple’s relationship had on the couple’s psychological and physical well-being. Partners experienced a range of conflicting emotions, which were largely due to changes in their partner’s behaviors and language (Li and Loke 2014). Individuals with MND often experience apathy, egocentrism, impulsivity, and decreased social adaptation, which can negatively impact caregivers (Rusina et al. 2021). Additionally, due to physical impairments, there is a reduction in intimacy between partners (Olesen et al. 2022). Partners reported feeling emotional and psychological distress watching their loved one deteriorate, as well as conflicting emotions such as guilt, anger, and resentment, which can have a negative effect on the couple’s mental well-being (Olesen et al. 2022). Behavioral changes as a result of MND are the strongest predictor of psychological distress in caregivers of individuals with MND (De Wit et al. 2019; Olesen et al. 2022). Partners also reported a decline in their physical health due to the physical demands and lack of rest that results from caring for an individual with MND. These findings are supported by research which suggests that partners have a reciprocal influence on each other’s quality of life and psychological health. Thus, changes in the couple’s relationship and role can have a negative effect on the couple’s mental and physical well-being, and vice versa (Baucom et al. 2020; Li and Loke 2014).

A final key theme that emerged from the review was the changes to social relationships external to the couple following an MND diagnosis and over the course of the disease trajectory. Several studies reported how partners of individuals with MND felt lonely and trapped within their relationship as they were no longer able to socialize as they once had (Wu et al. 2022). This is largely due to the time-consuming nature of caring for a partner with MND, which leaves little time for hobbies and social engagements (Wu et al. 2022). Studies also suggested that due to behavioral and cognitive changes, partners often felt embarrassed or awkward in public spaces for fear of their partner displaying inappropriate behaviors (Olesen et al. 2022). Thus, having a partner with MND affects how the couple relates to others (Olesen et al. 2022). This is supported by research that has found that partners who act as carers experience a sense of social isolation (Andréasson et al. 2023). Love et al. (2005) indicated that prolonged caring for an individual with MND results in a substantial loss of social support, which negatively affects the carer’s well-being, and may result in anxiety, depression, and psycho-social distress. This can lead to role dysfunction which ultimately negatively impacts their ability to provide care to the individual with MND and their relationship with their partner (Love et al. 2005).

## Future research

The findings of this research suggest that partners are integral to the care of individuals with MND (Flemming et al. 2020). However, despite the literature suggesting that caring for a partner with MND is likely to have a negative influence on the couple’s relationship, little support is available to partners of individuals with MND or for couples to assist them in planning for and navigating changes (Bilenchi et al. 2022; Flemming et al. 2020; Trucco et al. 2024). The main support available currently appears to be respite care. Yet, as the results of this review found, partners are often hesitant to use respite care as they are unwilling to share the burden of care due to a belief that the couple’s relationship is inviolable and that care is personal (Olesen et al. 2022). Therefore, it is important for future research to identify additional ways of supporting partners. Furthermore, the findings of this review suggest that future research needs to investigate ways of combating the social isolation that couples living with MND face (Andréasson et al. 2023). Finally, the results of the scoping review suggest that there is a lack of research on the impact of MND progression on intimacy between partners, with the suggestion that it is often a taboo subject and therefore not openly discussed (Flemming et al. 2020). However, given that shared love and intimacy are vital in reinforcing a strong couple relationship throughout the MND trajectory, future research needs to look at ways of supporting couples through these changes (Bassola et al. 2019). The National Institute for Health and Care Excellence (NICE) MND guidance suggests it is vital that health-care professionals discuss with individuals how the disease is likely to affect their daily living including adjusting to changes in relationships, roles, and intimacy (NICE 2016/2019). Therefore, future research is needed to develop a better understanding of how couple relationships change over the course of the disease trajectory, as well as what is needed to support partners through these relationship changes. This can guide future policies to help provide targeted support for couples and to gather evidence of what is to be expected to enhance the NICE guidance for conducting multidisciplinary team assessments. Such support and changes in policy might be able to improve the quality of life for both the person with MND and their partner, which is considered an urgent priority in the UK today (Kluger et al. 2023).

## Limitations

This study is not without limitations. First, several of the studies reported on informal carers or family carers or did not specifically report what relationships the carers had with the individual with MND. Very few studies reported entirely on the experiences of partners. As such, we were unable to derive from the data the experiences of partners exclusively, which may reduce the validity of the findings. Second, in comparison with the rigor of a systematic literature review, scoping reviews are less comprehensive and might render the study more vulnerable to bias. As observed by Tricco et al. (2016), there has been a marked increase in scoping reviews since 2012, but there remains variability in the ways in which scoping reviews are conducted and reported. In the case of this study, the research team attempted to minimize bias by following the PRISMA-ScR and ensuring that the screening procedure was reviewed by 2 members of the research team. Moreover, despite the potential limitations, a scoping review was deemed to be the most appropriate method in this case, given that the aim was to gain an understanding of the breadth of studies available in the apparent absence of any pre-existing comprehensive review of the impact of the MND journey on the couple’s relationship. This helped identify gaps in the existing literature, which might be a useful focus of research in this area.

## Conclusion

The results of this scoping review revealed that recieving an MND diagnosis and the subsequent progression of the disease has a profound impact on the couple’s relationship. The results suggest that MND can lead to changes in couple relationships through partners having to adjust to new roles, changes in communication, declines in caregivers’ health and well-being, changes in intimacy, and changes to social relationships. Future research is needed to develop a comprehensive understanding of how couple relationships change over the course of the MND trajectory and to guide future policies that will help provide targeted support to couples as they navigate these complex and challenging relationship changes.

## Supporting information

Malloy et al. supplementary material 1 Malloy et al. supplementary material

Malloy et al. supplementary material 2 Malloy et al. supplementary material

## Supplementary material

The supplementary material for this article can be found at https://doi.org/10.1017/S1478951524002141.
