# Supplementary material for: What is the current state of the research literature examining the impact of the motor neurone disease journey on the couple’s relationship? A scoping review
Source: Palliat Support Care. 2025 Mar 31;23:e85. doi: 10.1017/S1478951524002141 (PMC13166447; doi:10.1017/S1478951524002141)
Supplement: Malloy et al. supplementary material 1 — Malloy et al. supplementary material [file S1478951524002141sup001.docx]

**Appendix**

**National Centre for Social Research Framework Steps (Ritche and Spencer, 2002)**

| **Stage** | **Description** |
| --- | --- |
| **Stage 1.** Familiarization | Immersion in the data by reading and re-reading the texts to develop an overall sense of the data |
| **Stage 2.** Coding | Applying labels to describe the data |
| **Stage 3.** Developing a working analytical framework | Researchers compare and agree on coding, grouping the codes into categories to form an analytical framework |
| **Stage 4.** Applying the analytical framework | Framework is applied to data |
| **Stage 5.** Charting data | Data is summarized and restructured by categories |
| **Stage 6.** Interpreting the data | Data is analyzed and interpreted to develop deeper insights into the data |
